# Potential of *Aedes albopictus* to cause the emergence of arboviruses in Morocco

**DOI:** 10.1371/journal.pntd.0006997

**Published:** 2019-02-14

**Authors:** Fadila Amraoui, Wiem Ben Ayed, Yoann Madec, Chafika Faraj, Oumnia Himmi, Ameur Btissam, Mhammed Sarih, Anna-Bella Failloux

**Affiliations:** 1 Institut Pasteur, Department of Virology, Arboviruses and Insect Vectors, Paris, France; 2 Laboratory of Epidemiology and Veterinarian microbiology, Medical entomology, Tunis-Belvédère, Tunisia; 3 Institut Pasteur, Department of Infection and Epidemiology, Epidemiology of Emerging Diseases, Paris, France; 4 Laboratoire d'Entomologie Médicale, Institut National d’Hygiène, Rabat, Morocco; 5 Geophysics, Natural Patrimony and Green Chemistry Research Center, Geo-Biodiversity and Natural Patrimony Laboratory, Scientific Institute, Mohammed V Agdal University, Rabat, Morocco; 6 Service de lutte Anti-vectorielle, Direction de l’Epidémiologie et de Lutte contre les Maladies, Rabat, Morocco; 7 Service de Parasitologie et des Maladies Vectorielles, Institut Pasteur du Maroc, Casablanca, Morocco; University of Heidelberg, GERMANY

## Abstract

In 2015, the mosquito *Aedes albopictus* was detected in Rabat, Morocco. This invasive species can be involved in the transmission of more than 25 arboviruses. It is known that each combination of mosquito population and virus genotype leads to a specific interaction that can shape the outcome of infection. Testing the vector competence of local mosquitoes is therefore a prerequisite to assess the risks of emergence. A field-collected strain of *Ae*. *albopictus* from Morocco was experimentally infected with dengue (DENV), chikungunya (CHIKV), zika (ZIKV) and yellow fever (YFV) viruses. We found that this species can highly transmit CHIKV and to a lesser extent, DENV, ZIKV and YFV. Viruses can be detected in mosquito saliva at day 3 (CHIKV), day 14 (DENV and YFV), and day 21 (ZIKV) post-infection. These results suggest that the local transmission of these four arboviruses by *Ae*. *albopictus* newly introduced in Morocco is a likely scenario.

**Trial registration:** ClinicalTrials.gov APAFIS#6573-201606l412077987v2.

## Author summary

The Asian tiger mosquito *Aedes albopictus* is responsible for the transmission of several arboviruses such as dengue and chikungunya viruses. In 30 to 40 years, it has extended its geographical distribution in both tropical and temperate regions of all continents. The species was first detected in September 2015, in Rabat, Morocco. Using experimental infections, we demonstrated that *Ae*. *albopictus* Morocco are competent to transmit zika and yellow fever viruses in addition to the transmission of dengue and chikungunya viruses. Our results are central to suggest developing the most effective national surveillance program and to designing the most suitable control strategy to avoid the mosquito spreading beyond its point of entry in Morocco.

## Introduction

Over the past decades, arboviruses caused acute emergences leading to global pandemics. Dengue viruses (DENV; family Flaviviridae, genus *Flavivirus*) are responsible for 390 million infections per year including 96 million symptomatic cases [1]. In 2005, chikungunya virus (CHIKV; family Togaviridae, genus *Alphavirus*) emerged outside Africa producing devastated outbreaks in all continents [2]. While its importance was underestimated, zika virus (ZIKV; family Flaviviridae, genus *Flavivirus*) hit Brazil in 2015 causing several million cases in the Americas [3] and severe unusual symptoms such as Guillain-Barré syndrome and congenital microcephaly. Despite the availability of an efficient vaccine 17D, yellow fever virus (YFV; family Flaviviridae, genus *Flavivirus*) continues to cause human fatalities in South America and Sub-Saharan Africa.

All four arboviruses share the same mosquito vectors: *Aedes aegypti* and *Aedes albopictus*. *Ae*. *aegypti* is an urban mosquito feeding exclusively on humans [4] and *Ae*. *albopictus* colonizes a larger range of sites and feeds on both animals and humans [5]. While *Ae*. *aegypti* took several centuries to invade most countries in the world [6], *Ae*. *albopictus* took only a few decades to establish stable colonies worldwide [7]. Native to Southeast Asia, *Ae*. *albopictus* has invaded America, Africa and Europe during the last 40 years [8]. In Europe, it was introduced in 1979 in Albania and then in Italy in 1990. It is now present in 20 European countries [9]. In Africa, *Ae*. *albopictus* was first reported in the early 1990s in South Africa [10] and Nigeria [11]. Thereafter, it was described in several West and Central African countries: Cameroon in 2000 [12], Equatorial Guinea in 2003 [13], Gabon in 2007 [14], Central African Republic in 2009 [15], and Republic of Congo in 2011 [16]. More recently, it was detected in Mali [17], Mozambique [18] and São Tomé and Príncipe [19]. In North Africa, *Ae*. *albopictus* was detected in Algeria in 2010 [20] then in Morocco in 2015 [21].

Morocco is considered a low prevalent country for mosquito-borne diseases [22]. However, since 1996, the country has faced West Nile virus (WNV) with three epizootic episodes: 1996, 2003 and 2010 [23, 24]. In 2008, a serosurvey of wild birds confirmed the circulation of WNV in native birds [25]. Other arboviruses like Usutu virus and Rift valley fever virus (RVFV) have never been reported despite serological evidence of RVFV antibodies in camels at the border between Morocco and Mauritania [25–27]. Morocco is considered by several reports of the Intergovernmental Panel on Climate Change (IPCC) as a hotspot for climate change with its significant impact for several infectious diseases [28]. The introduction of an invasive species such as *Ae*. *albopictus* will likely cause a new public health problem. Moreover, Morocco is a tourist destination with more than 11 million visitors reported in 2017 {http://www.tourisme.gov.ma/fr/tourisme-en-chiffres/chiffres-cles}, increasing the risk of importing arboviral pathogens.

In this work, we evaluate the ability of *Ae*. *albopictus* recently introduced in Morocco to transmit CHIKV, DENV, ZIKV and YFV, where the outcome of vector infection depends on specific genotype-by-genotype (G x G) interactions between a vector population and a pathogen lineage [29]. This measure of the vector competence of field-collected mosquitoes helps to assess the risk of arbovirus emergence.

## Materials and methods

### Ethic statements

Animals were housed in the Institut Pasteur animal facilities accredited by the French Ministry of Agriculture for performing experiments on live rodents. Work on animals was performed in compliance with French and European regulations on care and protection of laboratory animals (EC Directive 2010/63, French Law 2013–118, February 6th, 2013). All experiments were approved by the Ethics Committee #89 and registered under the reference APAFIS#6573-201606l412077987 v2.

### Mosquito collections

During the national surveillance plan implemented in 2016 to establish the geographical distribution of *Ae*. *albopictus* in Morocco, five ovitraps less than 500 m apart were placed on a street of the Agdal neighborhood in Rabat (33°59'20.9′′ N, 6°51′07.9′′W). Ovitraps were checked for eggs once a week from May to November 2016 and were brought back to the laboratory to be stored in humid chambers (relative humidity of 80%) before being sent to Institut Pasteur in Paris to perform the vector competence studies.

After hatching, larvae were split into pans of 200 individuals and supplied every 2 days with a yeast tablet dissolved in 1L of dechlorinated tap water. All immature stages were reared at 26±1°C. Emerging adults were maintained at 28±1°C with a 16L:8D cycle, 80% relative humidity and supplied with a 10% sucrose solution. Females were fed twice a week on anaesthetized mice (OF1 mice, Charles River laboratories, France). Resulting F2 adults were used for vector competence assays. It should be noted that variations of oral susceptibility to an arbovirus can be considered negligible in fewer than five laboratory generations [30].

### Viral strains

CHIKV strain (06.21) was isolated from a patient on La Reunion Island in 2005 [31]. After isolation on *Ae*. *albopictus* C6/36 cells, this strain was passaged twice on C6/36 cells and the viral stocks produced were stored at -80°C prior to their use for mosquito oral infections. DENV-2 strain provided by Prof. Leon Rosen, was isolated from a human serum collected in Bangkok (Thailand) in 1974 [32] and had been passed only in different mosquito species (2 times in *Ae*. *albopictus*, 2 times in *Toxorhynchites amboinensis*, and one time in *Ae*. *aegypti*) by intrathoracic inoculation. Viral stocks were obtained by inoculating C6/36 cells. ZIKV strain (NC-2014-5132) originally isolated from a patient in April 2014 in New Caledonia was passaged five times on Vero cells; this strain belongs to the same genotype than the ZIKV strains circulating in Brazil in 2015 [33]. Lastly, a YFV strain (S79) belonging to the West African lineage, was isolated from a human case in Senegal in 1979 [34]. YFV-S79 was passaged twice on newborn mice and two times on C6/36 cells.

### Mosquito experimental infections

Six to eight batches of 60 7–10 day old females were exposed to an infectious blood meal containing 1.4 mL of washed rabbit erythrocytes and 700 μL of viral suspension. The blood meal was supplemented with ATP as a phagostimulant at a final concentration of 1 mM and provided to mosquitoes at a titer of 10^7.2^ plaque-forming unit (pfu)/mL for ZIKV, 10^6.5^ focus-forming unit (ffu)/mL for YFV and 10^7^ ffu/mL for CHIKV and DENV, using a Hemotek membrane feeding system. Mosquitoes were allowed to feed for 15 min through a piece of pork intestine covering the base of a Hemotek feeder maintained at 37°C. Fully engorged females were transferred in cardboard containers and maintained with 10% sucrose under controlled conditions (28±1°C, relative humidity of 80%, light:dark cycle of 16 h:8 h) for up to 21 days with mosquito analysis at 3, 7, 14 and 21 days post-infection (dpi). For each virus, 21–30 mosquitoes were examined at each dpi.

### Infection and dissemination assays

For each mosquito examined, body (abdomen and thorax) and head were tested respectively for infection and dissemination rates at 3, 7, 14 and 21 dpi. For this, each part was ground in 250 μL of Leibovitz L15 medium (Invitrogen, CA, USA) supplemented with 3% FBS, and centrifuged at 10,000×g for 5 min at +4°C. The supernatant was processed for viral titration.

### Transmission assays

Mosquitoes examined previously were also tested for viral transmission by collecting saliva using the forced salivation technique [35]. Mosquitoes were anesthetized on ice and legs and wings were removed. The proboscis was then inserted into a pipette tip containing 5 μL of fetal bovine serum (FBS). After 30 min, the tip content was transferred in 45 μL of L15 medium. Saliva was then titrated to estimate the transmission rate.

### Viral titration

CHIKV, DENV and YFV were titrated by focus fluorescent assay and ZIKV by plaque forming assay as ZIKV cannot produce distinct viral foci on mosquito cells.

### Focus forming assay on C6/36 cells

For mosquitoes challenged with CHIKV, DENV or YFV, saliva, head and body homogenates were titrated by focus fluorescent assay on *Ae*. *albopictus* C6/36 cells [36]. Samples were serially diluted and inoculated onto C6/36 cells in 96-well plates. After an incubation of 3 days for CHIKV, and 5 days for YFV and DENV-2 at 28°C, cells were stained using hyper-immune ascetic fluid specific to each virus as the primary antibody (CHIKV: provided by the French National Reference Center for Arbovirus at the Institut Pasteur, YFV: OG5 NB100-64510; Novusbio, CO, USA, and DENV: Ms X Dengue complex MAB 8705, Millipore, MA, USA) and Alexa Fluor 488 goat anti-mouse IgG (Life Technologies, CA, USA) as the secondary antibody. Saliva titers were expressed as ffu/saliva.

### Plaque forming assay on Vero cells

For ZIKV, body and head suspensions were serially diluted and inoculated onto monolayers of Vero cells in 96-well plates. Cells were incubated for 7 days at 37°C then stained with a solution of crystal violet (0.2% in 10% formaldehyde and 20% ethanol). Presence of viral particles was assessed by CPE detection. Saliva was titrated on monolayers of Vero cells in 6-well plates incubated 7 days under an agarose overlay. Saliva titers were expressed as pfu/saliva.

### Statistical analysis

Means, standard deviations, 95% confidence interval were calculated and statistical analyses were performed using the Stata software (StataCorp LP, Texas, and USA). The effect of virus and dpi on infection, dissemination and transmission rates was evaluated using Fisher’s exact test. The titer of viral particles in mosquito saliva was compared across groups using a Kruskall-Wallis non parametric test. P-values<0.05 were considered statistically significant. Heatmaps were built under R (v 3.3.1) (https://www.R-project.org).

## Results

### Viral infection

**Fig 1:**
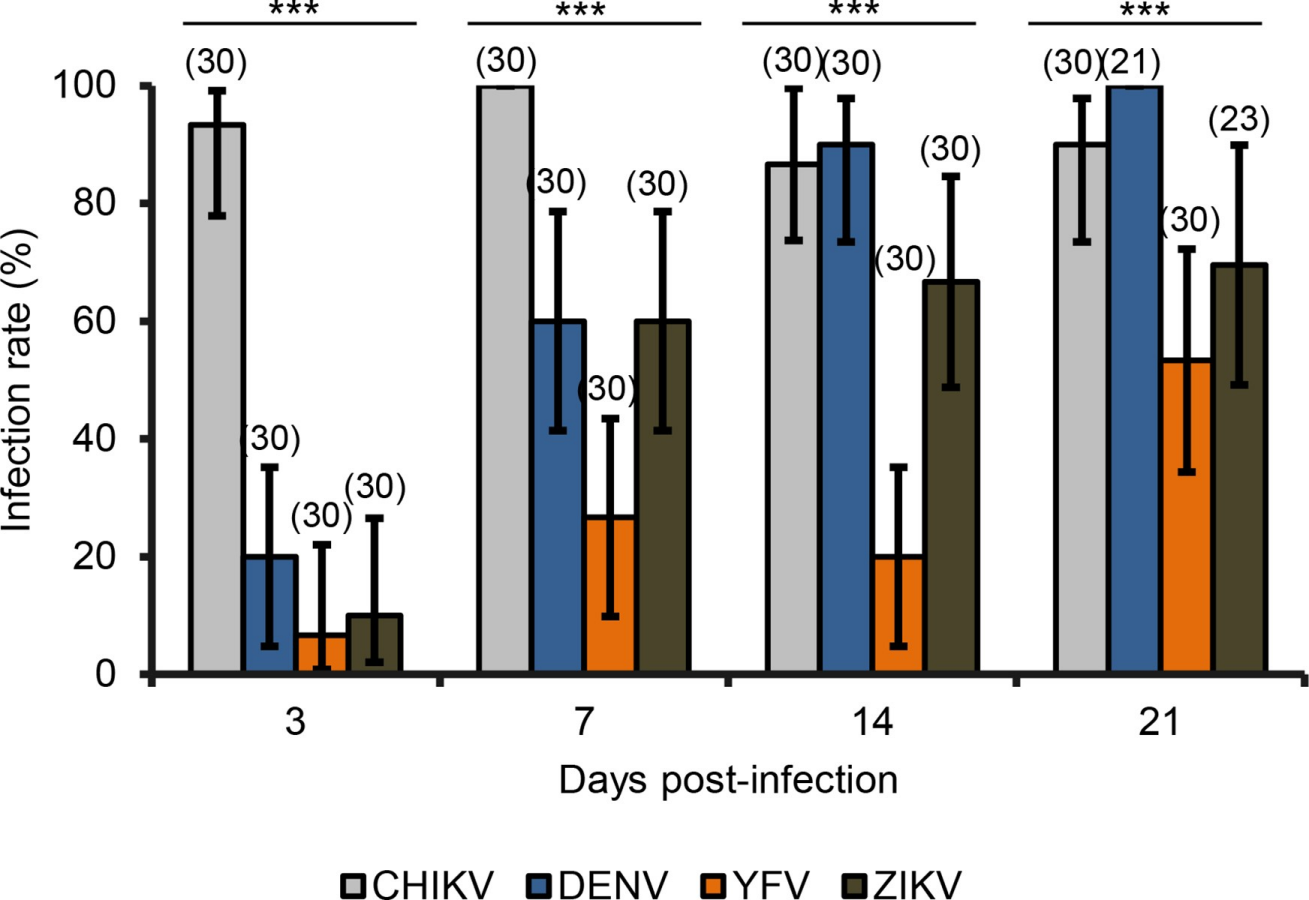
Infection rates of *Ae*. *albopictus* from Morocco with CHIKV, DENV, YFV, and ZIKV. F2 mosquitoes were orally challenged with CHIKV and DENV at a titer of 10^7^ ffu/mL, YFV at 10^6.5^ ffu/mL and ZIKV at 10^7.2^ pfu/mL. After infection, mosquito bodies were titrated at 3, 7, 14 and 21 days post-infection. Error bars showing the binomial 95% confidence interval (Stata software, StataCorp LP, Texas, and USA). In brackets, the number of mosquitoes examined. ***, P < 10^−4^.

Mosquito females were exposed to four separate infectious blood meals containing CHIKV, DENV, ZIKV or YFV. The first step after the ingestion of the infectious blood meal is the infection of the midgut which is appraised by calculating the infection rate (IR) corresponding to the proportion of mosquitoes with an infected midgut. At 3 dpi, *Ae*. *albopictus* Morocco were more infected with CHIKV (Fig 1; Fisher’s exact test: p<10^−4^, df = 3) with an IR reaching 93% (N = 30) whereas with the 3 other viruses, IRs were lower than 20% (N = 30). At 7 dpi, the IR with CHIKV reached 100% (N = 30) and remained significantly lower with DENV (60%; N = 30), ZIKV (60%; N = 30) and YFV (26.7; N = 30) (Fisher’s exact test: p<10^−4^, df = 3). At 14 dpi, mosquitoes become more infected with DENV reaching 90% (N = 30) close to CHIKV (86.7%, N = 30) (Fisher’s exact test: p = 0.69, df = 3) but significantly higher than with ZIKV (66.7%, N = 30), and YFV (20%, N = 30) (Fisher’s exact test: p<10^−4^, df = 30). At 21 dpi, the same pattern was observed: IRs were higher with CHIKV (90%, N = 30) and DENV (100%, N = 21) than with ZIKV (69.6%, N = 23) and YFV (53.3%, N = 30) (Fisher’s exact test: p<10^−4^, df = 3). IRs with all viruses increased along with dpi except with CHIKV which remained high (>86%) very early from 3 dpi. The lowest IRs were obtained with YFV fluctuating from 6.7% at 3 dpi to 53.3% at 21 dpi.

### Viral dissemination

**Fig 2:**
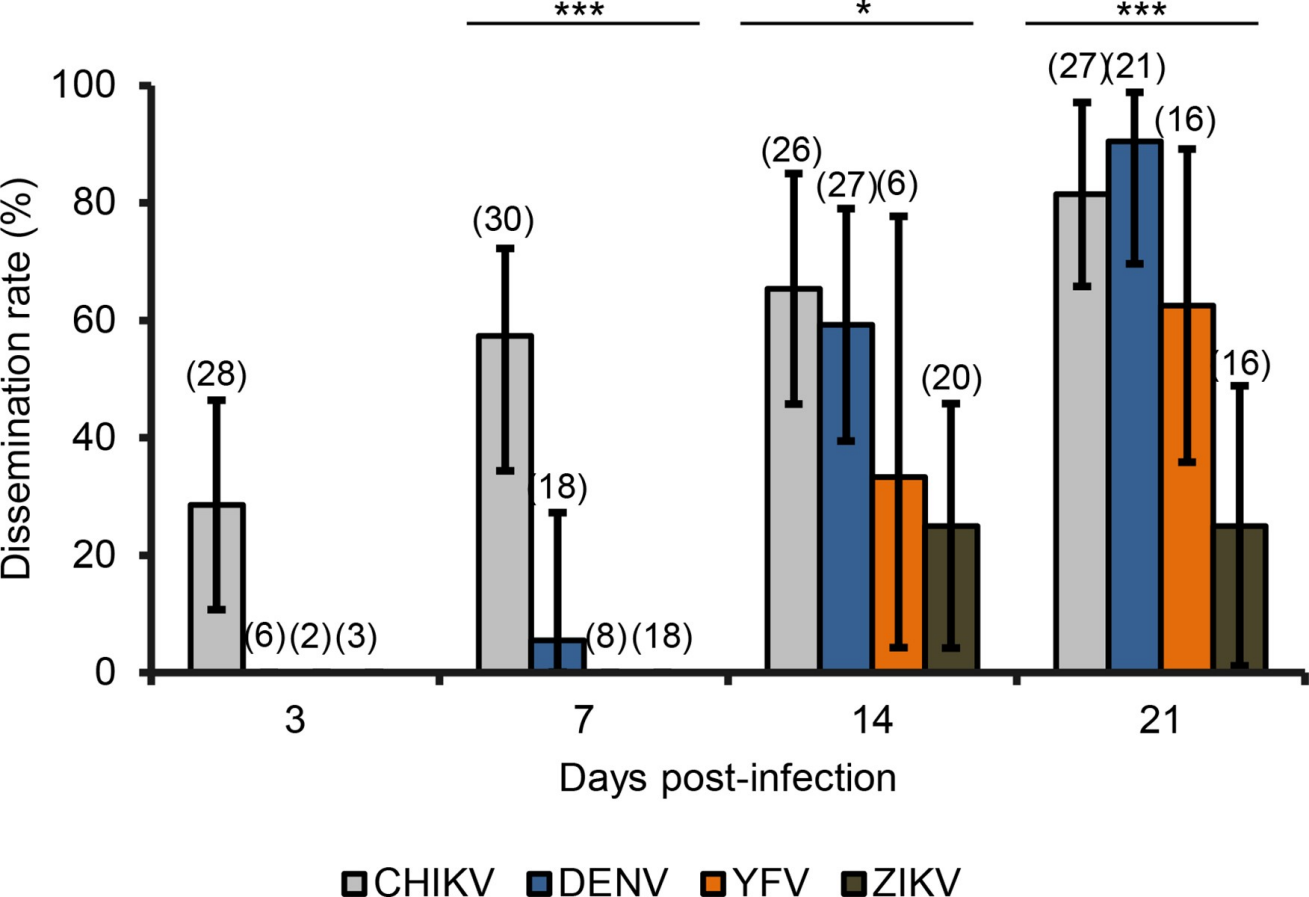
Dissemination rates of *Ae*. *albopictus* from Morocco with CHIKV, DENV, YFV, and ZIKV. After infection, mosquito heads were titrated at 3, 7, 14 and 21 days post-infection. Error bars showing the binomial 95% confidence interval (Stata software, StataCorp LP, Texas, and USA). In brackets, the number of mosquitoes examined. *, P < 0.05***, P < 10^−4^.

Once the midgut is infected, viral particles can disseminate from the midgut to internal organs and tissues. The dissemination rate (DR) gives the number of mosquitoes with infected heads among mosquitoes with infected midgut. At 3 dpi, only CHIKV was detected in mosquito heads (Fig 2; 28.6%, N = 28). At 7 dpi, DR with CHIKV reached 53.3% (N = 30) and only 5.5% (N = 18) with DENV (Fisher’s exact test: p<10^−4^, df = 3). At 14 dpi, DRs with CHIKV (65.4%, N = 26) and DENV (59.2%, N = 27) were higher and similar (Fisher’s exact test: p = 0.65, df = 1) compared to YFV (33.3%, N = 6) and ZIKV (25%, N = 20) which were both lower and comparable (Fisher’s exact test: p = 0.69, df = 1). At 21 dpi, DRs for each virus were significantly different (Fisher’s exact test: p<10^−4^, df = 3) and slightly higher than the DRs at 14 dpi. Viral dissemination started earlier with CHIKV at 3 dpi while it was only at 7 dpi with DENV and 14 dpi with YFV and ZIKV. The lowest DRs were obtained with ZIKV maintained at 25% at 14 and 21 dpi.

### Viral transmission

**Fig 3:**
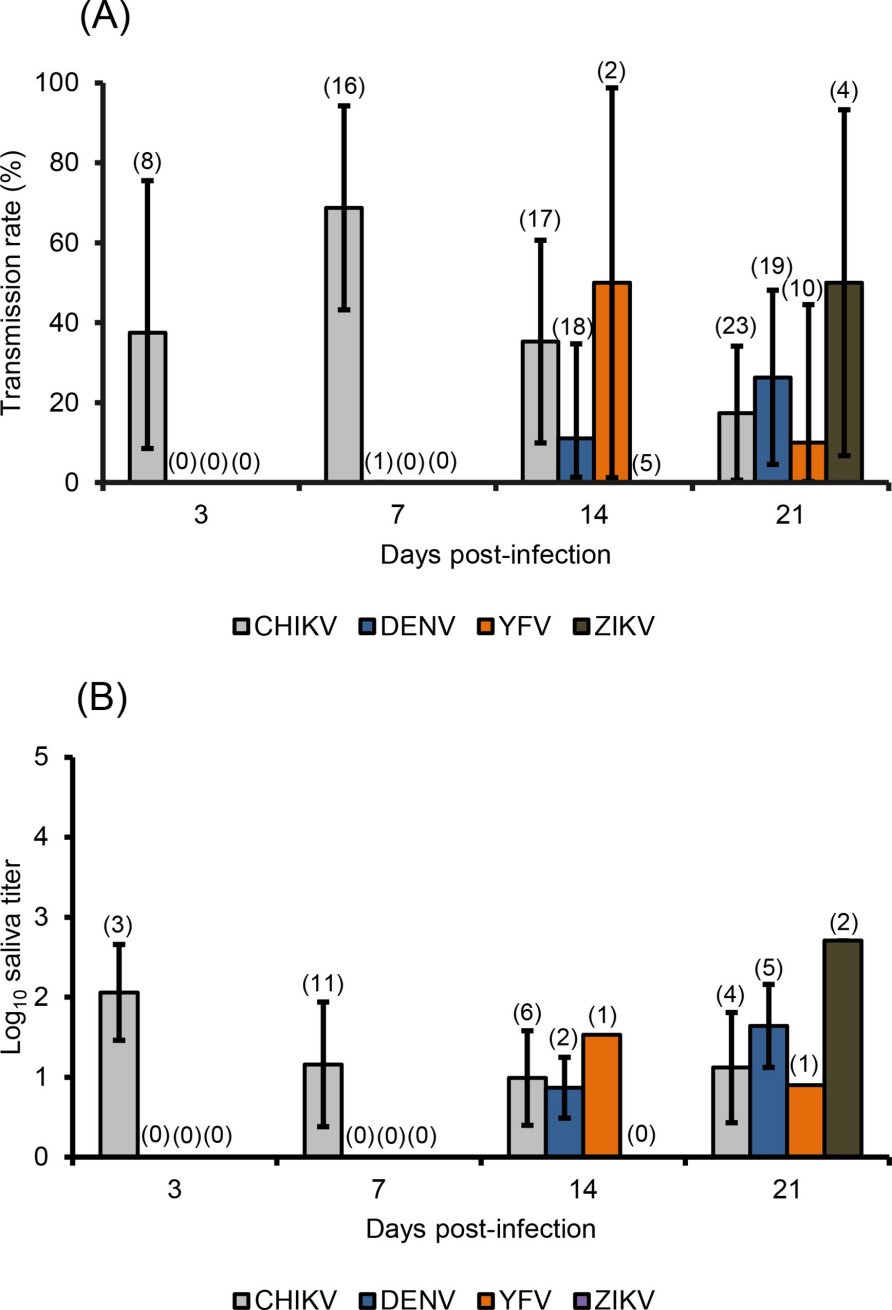
**Transmission rates (A) and mean titer of infectious viral particles in saliva (B) of *Ae*. *albopictus* from Morocco infected with CHIKV, DENV and YFV, and ZIKV.** Saliva was collected from surviving females using the forced salivation technique at 3, 7, 14 and 21 days post-infection. Error bars show the binomial 95% confidence interval in (A) and the standard deviation in (B), both calculated using the Stata software (StataCorp LP, Texas, and USA). In brackets, the number of mosquitoes examined.

After the virus has spread into the general cavity of the mosquito and infected the salivary glands, the virus must be excreted in saliva for subsequent transmission. The transmission rate (TR) is defined as the proportion of mosquitoes delivering infectious saliva among mosquitoes having disseminated the virus (Fig 3A). At 3 and 7 dpi, viral particles could be detected in saliva of mosquitoes infected with CHIKV, with TRs of 37.5% (N = 8) and 68.7% (N = 16) respectively. At 14 dpi, TR with YFV (50%, N = 2) predominated over TRs with CHIKV (35.3%, N = 17) and DENV (11.1%, N = 18), TR with ZIKV remaining at 0%; no significant difference was observed among all TRs (Fisher’s exact test: p = 0.14, df = 3). At 21 dpi, transmission with ZIKV became detectable with a TR of 50% (N = 4), not significantly different from TRs with DENV (26.3%, N = 19), CHIKV (17.4%, N = 23), and YFV (10%, N = 10) (Fisher’s exact test: p = 0.36, df = 3). Transmission started early at 3 dpi with CHIKV, at 14 dpi with DENV and YFV, and at 21 dpi with ZIKV with respectively, a mean of 2.06±0.60 Log_10_ ffu/saliva (N = 3), 0.87±0.38 Log_10_ ffu/saliva (N = 2), 1.53 Log_10_ ffu/saliva (N = 1), and 2.71±0.01 Log10 pfu/saliva (N = 2) (Fig 3B). No significant difference was detected between all viruses at 14 dpi (Kruskal-Wallis test: p = 0.47, df = 2) and 21 dpi (Kruskal-Wallis test: p = 0.10, df = 3). The highest number of viral particles was detected in saliva of mosquitoes infected with YFV and examined at 21 dpi: TR of 50% (2 among 4 mosquitoes with viral dissemination), 2 females delivering 2.70 Log10 pfu (500) and 2.72 Log10 pfu (530) infectious particles.

### Transmission efficiency

**Fig 4:**
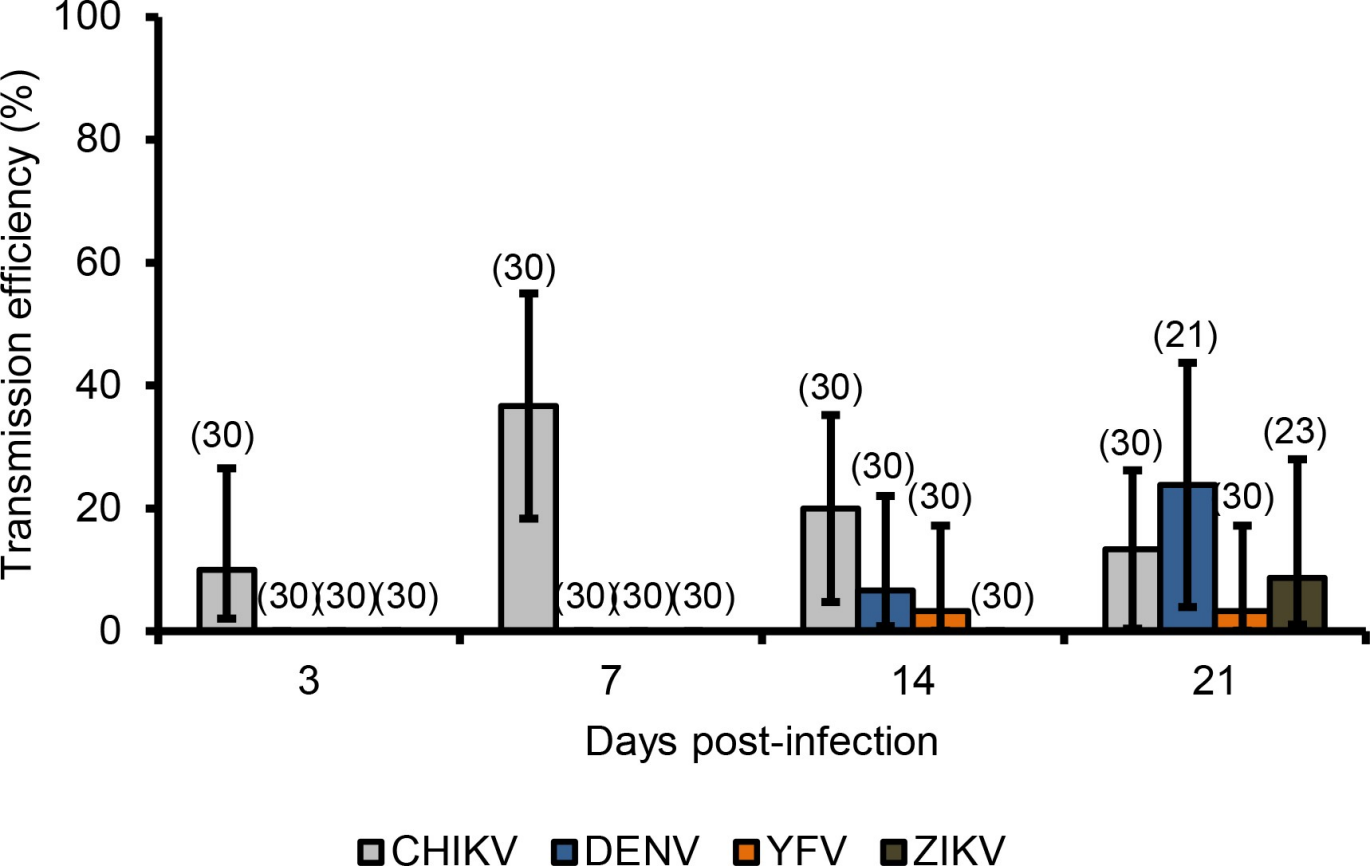
Transmission efficiencies of *Ae*. *albopictus* from Morocco infected with CHIKV, DENV, YFV, and ZIKV. Saliva was collected from surviving females at 3, 7, 14 and 21 days post-infection. Transmission efficiency was calculated as the proportion of mosquitoes with infectious saliva among mosquitoes initially tested. Error bars showing the binomial 95% confidence interval (Stata software, StataCorp LP, Texas, and USA). In brackets, the number of mosquitoes examined.

Whereas IR, DR and TR measure the efficiency of the midgut and salivary glands barriers to modulate, respectively, viral dissemination and transmission, the transmission efficiency (TE) gives an overview of transmission potential of mosquitoes tested; it corresponds to the proportion of mosquitoes with infectious saliva among all mosquitoes examined (presenting or not a viral dissemination with infected heads). Fig 4 shows that, the highest TE was detected at 7 dpi with CHIKV, at 21 dpi with DENV, at 14/21 dpi with YFV, and at 21 dpi with ZIKV. Collectively, *Ae*. *albopictus* Morocco were more susceptible to CHIKV and secondarily, to DENV, ZIKV and YFV.

### Vector competence

**Fig 5:**
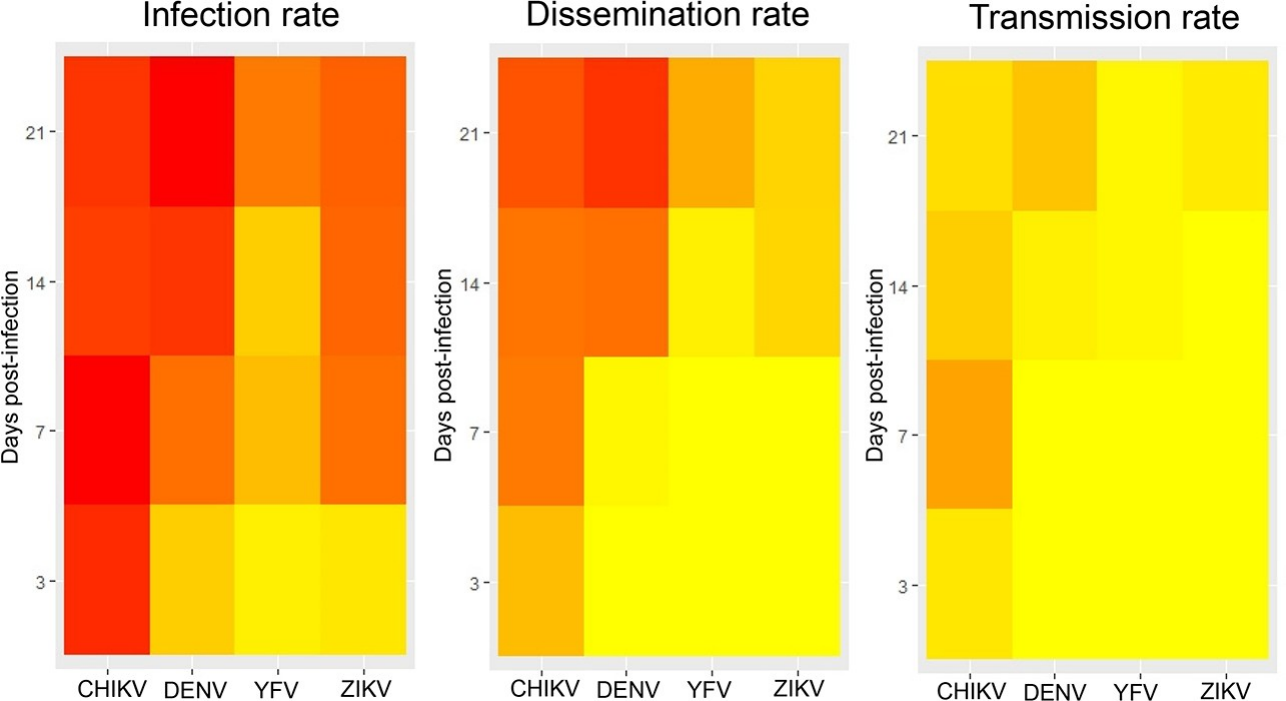
Heatmaps indicating interactions between viruses (DENV, CHIKV, YFV, ZIKV) and days post-infection for infection, dissemination and transmission. The intensity of red increases with the rates.

To summarize the vector competence corresponding to the overall ability of a mosquito population to be infected, to ensure the viral dissemination and to transmit the virus, heatmaps were built (Fig 5). *Ae*. *albopictus* Morocco were better infected with CHIKV from 3 dpi than with DENV and ZIKV (Fig 5A). Mosquitoes ensured an early dissemination (Fig 5B) and transmission (Fig 5C) with CHIKV (from 3 dpi) than with DENV and ZIKV. The species was less susceptible to YFV. Altogether, vector competence of *Ae*. *albopictus* Morocco depends on the virus and the dpi: it is more susceptible to CHIKV and susceptibility increases along with the dpi.

## Discussion

Using experimental infections, we show that the recently-introduced *Ae*. *albopictus* in Morocco were susceptible to all four viruses tested, CHIKV, DENV, YFV and ZIKV. Viral transmission was detected at 3 dpi with CHIKV, 14 dpi with DENV and YFV, and only 21 dpi with ZIKV.

Even if DENV, YFV and ZIKV belong to the same genus, they behave differently in *Ae*. *albopictus* mosquitoes. Infection of the midgut increases gradually from 3 dpi: DENV infects more efficiently mosquitoes than YFV and ZIKV, YFV remaining the less successful. Dissemination of DENV from the midgut to the mosquito general cavity started at 7 dpi as observed with most populations of *Ae*. *albopictus* [37]; it takes a shorter time with *Ae*. *aegypti*, i.e. from 5 dpi [38]. DENV dissemination is more strongly inhibited at early dpi than later meaning that the role of midgut as a barrier is diminished with dpi. Transmission of DENV was observed from 14 dpi suggesting an intrinsic incubation period higher than 7 dpi, likely around 10 dpi [37]. With ZIKV and YFV, dissemination was observed only at 14 dpi, YFV spreading at a higher rate than ZIKV suggestive of a stronger role of the midgut barrier with YFV. Transmission was detected at 14 dpi with YFV as observed with other *Ae*. *albopictus* populations [39] and 21 days with ZIKV which is longer than expected [40].

CHIKV presents a different profile. This alphavirus infects, disseminates and is transmitted more intensively and more quickly than the three other viruses. This viral strain presents an amino acid substitution (A226V) in the envelope glycoprotein E1 [31] favoring the viral transmission by *Ae*. *albopictus* [41, 42]. Importantly, exposure of infected mosquitoes to lower temperatures (lower than 25°C) compatible to values recorded in Morocco can modulate transmission [37]. It has been demonstrated that *Ae*. *albopictus* were able to better transmit CHIKV at a temperature lower than 28°C [43].

These assessments of vector competence of *Ae*. *albopictus* from Morocco to CHIKV, DENV, ZIKV and YFV are important for appraising the risk of local transmission. ZIKV shows the longer extrinsic incubation period (EIP) which refers to the time between the uptake of the virus during the blood feeding and the delivery of the virus by vector bite after successful infection and dissemination in the mosquito. If the EIP is longer than the daily survival rate of the mosquito, the risk of transmission is low. By shortening mosquito lifespan, vector control measures reduce disease transmission [44]. However, other factors such as environmental factors, e.g. the temperature, may influence the vector competence [43]. The vector competence and the EIP both contribute to estimating the vector capacity which describes the basic reproductive rate of a pathogen by a vector [44]. A high abundance of the vector [45], increased contacts between the vector and humans (i.e. anthropophily of mosquitoes) [5] and a high proportion of immunologically naïve humans, are also factors that should be considered in estimating the risk of emergence. Introductions of viremic travelers from endemic countries for all these viruses may initiate local transmission and outbreaks. Therefore surveillance of travelers must be reinforced.
